# Mitochondrial CHCHD2: Disease-Associated Mutations, Physiological Functions, and Current Animal Models

**DOI:** 10.3389/fnagi.2021.660843

**Published:** 2021-04-22

**Authors:** Teresa R. Kee, Pamela Espinoza Gonzalez, Jessica L. Wehinger, Mohammed Zaheen Bukhari, Aizara Ermekbaeva, Apoorva Sista, Peter Kotsiviras, Tian Liu, David E. Kang, Jung-A. A. Woo

**Affiliations:** ^1^USF Health Byrd Alzheimer’s Center and Research Institute, Tampa, FL, United States; ^2^Department of Molecular Pharmacology and Physiology, USF Health Morsani College of Medicine, Tampa, FL, United States; ^3^Department of Molecular Medicine, USF Health Morsani College of Medicine, Tampa, FL, United States; ^4^James A. Haley Veterans Administration Hospital, Tampa, FL, United States

**Keywords:** CHCHD2, CHCHD10, mitochondria, Parkinson’s disease, Lewy body disorders

## Abstract

Rare mutations in the mitochondrial protein coiled-coil-helix-coiled-coil-helix domain containing 2 (CHCHD2) are associated with Parkinson’s disease (PD) and other Lewy body disorders. CHCHD2 is a bi-organellar mediator of oxidative phosphorylation, playing crucial roles in regulating electron flow in the mitochondrial electron transport chain and acting as a nuclear transcription factor for a cytochrome c oxidase subunit (COX4I2) and itself in response to hypoxic stress. CHCHD2 also regulates cell migration and differentiation, mitochondrial cristae structure, and apoptosis. In this review, we summarize the known disease-associated mutations of CHCHD2 in Asian and Caucasian populations, the physiological functions of CHCHD2, how CHCHD2 mutations contribute to α-synuclein pathology, and current animal models of CHCHD2. Further, we discuss the necessity of continued investigation into the divergent functions of CHCHD2 and CHCHD10 to determine how mutations in these similar mitochondrial proteins contribute to different neurodegenerative diseases.

## Introduction

Lewy body pathology, in the form of intracellular Lewy bodies (LBs) and Lewy neurites (LNs), represents the second most common brain proteinopathy (Wakabayashi et al., 2013). LBs and LNs are found in both subcortical and cortical brain regions of the spectrum of Lewy body disorders (LBDs). LBDs include Parkinson’s disease (PD), Parkinson’s disease dementia (PDD), Dementia with Lewy bodies (DLB), and Multiple system atrophy (MSA). The major component of Lewy body pathology is α-synuclein (Jellinger, 2018), an abundant presynaptic protein that misfolds and builds up in toxic assemblies to cause neurodegeneration in LBDs (Spillantini et al., 1997).

PD is the second most common neurodegenerative disease affecting ∼1% of people over the age of 60 and is associated with motor symptoms such as tremor, bradykinesia, and changes in posture and speech (Jankovic, 2008; Ryan et al., 2015). Non-motor symptoms include neuropsychiatric dysfunction, sleep disorders, autonomic dysfunction, sensory symptoms, and pain. Therefore, patients develop loss of smell or taste, depression, sleep problems, hallucinations and genitourinary dysfunction (Chaudhuri et al., 2006; Poewe, 2008). While most PD cases are sporadic, approximately 5–10% of PD patients have a positive family history and tend to present with earlier disease onset (Ryan et al., 2015). While the buildup of misfolded α-synuclein and ubiquitin protein aggregates in LBs is associated with PD symptoms and death of dopaminergic (DA) neurons in the substantia nigra (Spillantini et al., 1997), the mechanistic basis of α-synuclein buildup and its toxicity leading to DA neuron death remains to be fully elucidated.

Parkinson’s disease dementia (PDD) is another type of LBD, characterized by the presence of PD symptoms along with dementia (Burton et al., 2004). Patients with PD have a 6-fold increased risk of developing dementia compared to healthy controls (Aarsland et al., 2001; Burton et al., 2004). Pathologically, PD patients show gray matter loss in frontal brain regions while PDD patients show gray matter loss in temporal, occipital, and subcortical areas (Burton et al., 2004), although both diseases typically affect the brainstem (Kosaka, 2014). DLB is the third most common form of dementia after Alzheimer’s Disease (AD) and vascular dementia (Jellinger, 2018; Urbizu and Beyer, 2020). DLB affects 1 to 2 percent of the population (Urbizu and Beyer, 2020), specifically having a higher incidence and prevalence in populations over 65 years old (Shiner et al., 2016). It is primarily sporadic and shares genetic risk determinants with PD and AD (Selikhova et al., 2009). Clinical features of DLB include three categories: cognitive impairment, behavioral/psychiatric phenomena, and physical symptoms (Donaghy and McKeith, 2014). Another distinguishing feature of DLB is a higher load of cortical Lewy body pathology in the temporal and parietal brain regions (Masliah, 2001; Selikhova et al., 2009). While DLB and PDD are synucleinopathies with similar incidence rates (Jellinger, 2018; Urbizu and Beyer, 2020), differences arise in the order of symptom onset. Patients with PDD develop motor symptoms first and then later show symptoms of dementia, while patients with DLB are diagnosed with dementia and then later show motor impairments (Burton et al., 2004; Kosaka, 2014). Notably, around 30% of PD patients and up to 50% of LBD and PDD patients have enough amyloid beta plaques and neurofibrillary tangle pathology to have a secondary diagnosis of AD (Irwin et al., 2012, 2013; Irwin and Hurtig, 2018). Furthermore, LBD patients with increasing AD pathologies have higher cerebral α-synuclein scores, earlier dementia onset, and shorter disease duration (Irwin et al., 2017), demonstrating the common co-occurrence of multiple pathologies in LBDs. Up to 70% of DLB patients at autopsy also show medium to high AD pathologic change (Irwin et al., 2017), demonstrating the common co-occurrence of multiple pathologies in LBDs. Indeed, the accumulation of amyloid beta enhances α-synuclein aggregation (Emmer et al., 2012; Koppen et al., 2020), and tau is required for synaptic and memory deficits in the α-synuclein A53T transgenic mouse model (Singh et al., 2019).

Mitochondria are multifunctional organelles that generate ATP (Brown, 1992) and regulate apoptosis (Kroemer, 1997; Green and Reed, 1998; Susin et al., 1999; Joza et al., 2001), calcium homeostasis (Gunter and Pfeiffer, 1990), and reactive oxygen species (ROS) production (Brand, 2010; Sena and Chandel, 2012; Palikaras et al., 2015). Healthy mitochondria are critical to neuronal homeostasis, as neurons have the highest energy demand among the cells in the brain. Multiple studies have shown that DA neurons are particularly vulnerable to oxidative stress and mitochondrial stressors due to higher energy requirements than other types of neurons for proper excitability and survival (Bolam and Pissadaki, 2012; Surmeier et al., 2017; Mamelak, 2018). Increasing evidence suggests that mitochondrial dysfunction is a major component of neurodegenerative diseases (Beal, 1998; Lin and Beal, 2006; Johri and Beal, 2012). Under physiological conditions, unhealthy or damaged mitochondria undergo selective clearance by autophagy, specifically known as mitophagy. However, in neurodegenerative disease, mitophagy is disrupted (Ciechanover and Brundin, 2003), thereby leading to the accumulation of misfolded proteins and dysfunctional mitochondria (Chen and Chan, 2009). The accumulation of damaged mitochondria leads to cellular damage and death (Lin and Beal, 2006). Indeed, PD-linked recessive mutations in *PINK1* and *Parkin* are associated with mitochondrial dysfunction and defects in mitophagy, indicating that such mitochondrial pathogenesis can drive the PD phenotype (Matsumine et al., 1997; Hattori et al., 1998; Kitada et al., 1998; Bentivoglio et al., 2001; Valente et al., 2001, 2002).

Coiled-coil-helix-coiled-coil-helix domain containing 2 (CHCHD2/MNRR1) and coiled-coil-helix-coiled-coil-helix domain containing 10 (CHCHD10) are two homologous sister proteins, both of which are localized to the intermembrane space (IMS) of mitochondria to regulate mitochondrial homeostasis. In mice, knockout of either *CHCHD2* or *CHCHD10* produce little to no gross abnormalities (Burstein et al., 2018; Liu Y.T. et al., 2020; Ryan et al., 2021; Sato et al., 2021). Homozygous *CHCHD10*^–/–^ mice are viable with no bioenergetic defects or mitochondrial abnormalities in dopaminergic processes in the brain, heart, and skeletal muscle (Burstein et al., 2018). Interestingly, there was no difference in mitochondrial abnormalities in dopaminergic processes or other cell types between wild type and *CHCHD10*^–/–^ mice (Burstein et al., 2018). Homozygous *CHCHD2*^–/–^ mice are also viable and have a normal survival rate. However, *CHCHD2*^–/–^ mice exhibit p62 inclusions and dopaminergic neuronal loss in an age-dependent manner (Sato et al., 2021). Double knockout of both genes results in clear mitochondrial phenotypes and activation of the integrated mitochondrial stress response (Liu Y.T. et al., 2020). Despite multiple functional overlaps and 54% protein sequence identity between CHCHD2 and CHCHD10, rare mutations in *CHCHD2* are associated with PD and other LBDs (Funayama et al., 2015; Ogaki et al., 2015), whereas mutations in *CHCHD10* are associated with the spectrum of amyotrophic lateral sclerosis (ALS)-frontotemporal dementia (FTD) (Bannwarth et al., 2014; Johnson et al., 2014; Müller et al., 2014; Zhang et al., 2015). At present, human genetic evidence for *CHCHD2* in PD and LBD pathogenesis is compelling but not conclusive. In this review, we aim to focus on CHCHD2 and summarize disease-associated CHCHD2 mutations, known functions of CHCHD2, and current animal models of CHCHD2 with relevance to mitochondria and LBDs.

## Disease-Associated *CHCHD2* Mutations in Asian Populations

**FIGURE 1:**
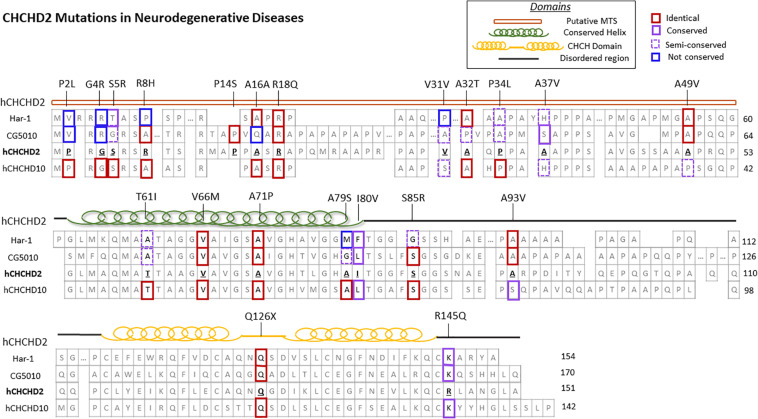
CHCHD2 amino acid sequence alignment and disease-associated mutations. Secondary structure and protein sequence alignment between human CHCHD2 and its homolog CHCHD10, along with their orthologs *Caenorhabditis elegans* har-1 and *Drosophila* CG5010. The amino acid positions of CHCHD2 disease-linked mutations are shown. The degree of conservation of affected residues in CHCHD2 versus homologous proteins are outlined in red (identical), purple (conserved), dashed purple (semi-conserved), and blue (not conserved).

Coiled-coil-helix-coiled-coil-helix domain containing 2, along with its homolog CHCHD10, belongs to the coiled-coil-helix-coiled-coil-helix (CHCH) domain protein family, characterized by two cysteine-X9-cysteine (CX_9_C) motifs (Imai et al., 2019b). Both proteins are evolutionarily conserved and single-copy orthologs *har-1* and *CG5010* are found in *Caenorhabditis elegans* and *Drosophila*, respectively (Figure 1; Imai et al., 2019b). These CX_9_C-containing proteins are imported into the mitochondrial IMS and regulate important physiological functions such as redox and mitochondrial respiration (Modjtahedi et al., 2016).

In the aftermath of the discovery of *CHCHD10* mutations in familial ALS-FTD patients (Bannwarth et al., 2014), Funayama and colleagues first reported two *CHCHD2* missense mutations (T61I and R145Q) and one splice-site mutation (300 + 5G > A) in late-onset autosomal dominant PD in Japanese families (Funayama et al., 2015). Specifically, the T61I and R145Q mutations were detected in 3 out of 340 Japanese autosomal dominant PD families, whereas neither mutation was found in 517 sporadic PD patients and 559 controls (Funayama et al., 2015). Later, the T61I mutation was identified in a Chinese family with autosomal dominant PD (Shi et al., 2016), and the R145Q mutation was identified in a sporadic Han Chinese PD patient (Yang et al., 2016), indicating that autosomal dominant parkinsonism could be caused by CHCHD2 mutations in Chinese populations.

Recent brain autopsy data from the original Japanese family carrying the CHCHD2 T61I mutation (Funayama et al., 2015) revealed widespread α-synuclein pathology with LBs present in the brain stem, neocortex, and limbic regions (Ikeda et al., 2019). Interestingly, this patient also exhibited amyloid plaques and neurofibrillary tangles. While the CHCHD2 aggregates were found to strongly colocalize with S129 phospho-α-synuclein positive LBs (Ikeda et al., 2019), reduced colocalization of CHCHD2 and mitochondria was observed in this patient compared to sporadic PD and control cases. These data suggest that the T61I mutation might impair the mitochondrial import of CHCHD2 (Ikeda et al., 2019). CHCHD10, which is known to physically interact with CHCHD2 (Burstein et al., 2018; Straub et al., 2018), was also found to be insolubilized by the T61I mutation (Ikeda et al., 2019). Biochemical analysis showed elevated S129 phospho-α-synuclein in the sarkosyl-soluble and insoluble fraction of the T61I patient brain (Ikeda et al., 2019). Furthermore, structural characterization of α-synuclein fibrils revealed the prion-like seeding activity of α-synuclein from T61I patients is similar to that of DLB and *SNCA* duplication patients (Ikeda et al., 2019). Overall, this study strongly suggests that a rare CHCHD2 mutation can contribute to the development of LB pathology. Additionally, the analogous structure of α-synuclein fibrils between *SNCA* duplication, DLB, and CHCHD2 T61I patients suggests the relevance of CHCHD2 in the development of LBDs. However, it is notable that these observations came from a single CHCHD2 T61I mutation carrier, thereby requiring further investigation from additional autopsy cases for validation.

Funayama et al. (2015) also identified the single nucleotide variants (SNVs) −9T > G and P2L (5C > T) in their Japanese study cohort (*n* = 517) of sporadic PD patients. While the −9T > G variant has also been observed in two other studies of PD patients in China (Lu et al., 2016; Wu et al., 2016), the frequency of this variant was not significantly different between the control and disease groups (Lu et al., 2016; Wu et al., 2016). The P2L variant (5C > T), which is positioned in the mitochondrial targeting sequence (MTS), is predicted to be pathogenic and to possibly affect CHCHD2 localization in the IMS (Funayama et al., 2015). P2L has been associated with sporadic PD in further studies in mainland China (Shi et al., 2016) and Chinese patients in Singapore (Foo et al., 2015). On the contrary, other studies in Taiwan and China did not find a significant difference in the frequency of the P2L variant between sporadic PD patients and the controls (Fan et al., 2016; Wu et al., 2016; Yang et al., 2016). This association from various ethnicities was later presented in a meta-analysis, suggesting that P2L may be a risk factor for sporadic PD in Asian populations (Yang et al., 2016).

Other notable variants of the *CHCHD2* gene have been found in further studies of neurodegenerative diseases in Japan and China. Another mutation in the MTS of CHCHD2 (R8H) was found in a sporadic PD patient in the Japanese island of Sado (Ikeda et al., 2017). The R8 residue in CHCHD2 is fully conserved across species from *Xenopus*, rodents, to humans and is predicted to be “probably damaging” or “damaging” by PolyPhen-2 and SIFT algorithms (Ikeda et al., 2017), respectively, although functional studies are required to validate its pathogenicity. The A79S mutation in the highly conserved hydrophobic helix of CHCHD2 was identified in a sporadic PD patient in China (Yang et al., 2019). CADD, PolyPhen-2, and SIFT algorithms also predicted this mutation to be pathogenic (Yang et al., 2019). Wu et al. (2016) reported the heterozygous *CHCHD2* variant *154A > G the 3′-untranslated region (3′-UTR) in two Chinese autosomal dominant PD patients, but not in the control group. Similarly, R18Q was found in a Han Chinese late-onset sporadic PD patient and is predicted as “probably damaging” and “damaging” by PolyPhen-2 and SIFT, respectively (Yang et al., 2016). To determine if rare *CHCHD2* mutations are associated with dementia, another study from mainland China screened 150 AD, 84 FTD, and 417 controls for the presence of *CHCHD2* mutations. Interestingly, four rare putative pathogenic variants of *CHCHD2* were identified in AD patients (P2L, S5R, and A32T) and FTD patients (S85R) (Che et al., 2018), which suggests that *CHCHD2* mutations may drive pathologies beyond Lewy bodies. Thus far, studies from Asian populations support a role for rare *CHCHD2* mutations in the development of PD and possibly other neurodegenerative diseases.

## Disease-Associated *CHCHD2* Mutations in Caucasian Populations

The vast majority of *CHCHD2* genetic variants observed in Caucasian populations are distinct from those observed in Asian populations. Specifically, of the 15 rare exonic *CHCHD2* genetic variants observed in Caucasian patients described below, only two of them (P2L and A32T) have been found in Asian populations. A large multicenter study from a United States Caucasian cohort (878 PD, 610 LBD, and 717 controls), an Irish cohort (355 PD and 365 controls), and a Polish cohort (394 PD and 350 controls) identified 9 rare exonic *CHCHD2* variants (Table 1): P2L, G4R, P14S, A16A (c.40C > T), V31V (c.93C > T), P34L, A37V, A49V, and A93V (Ogaki et al., 2015). These exonic variants were significantly overrepresented in PD and LBD patients compared to controls (0.6% vs. 0.1%), supporting a role for these *CHCHD2* variants as genetic risk factors for PD and LBD. Interestingly, 8 out of the 9 exonic variants observed in this study were in the MTS of CHCHD2 (Ogaki et al., 2015), suggesting the mistargeting of CHCHD2 as a potential disease mechanism.

**TABLE 1:** Disease-associated mutations of CHCHD2 in neurodegenerative diseases.

Mutation	Type of mutation	Location	Ethnicity	Sex ratio (Female: Male)	Age at onset (mean)	Predominant symptoms		Disease	References
						Motor	Non-motor		
P2L (c. 5C > T)	Heterozygous missense	MTS domain	Chinese	ND	ND	ND	ND	Early-onset PD and sporadic PD	Foo et al., 2015
			Chinese	ND	ND	ND	ND	Sporadic PD	Shi et al., 2016
			Japanese	ND	ND	ND	ND	Sporadic PD	Funayama et al., 2015
			Chinese	2:1	65.67	ND	ND	AD	Che et al., 2018
			Caucasian	DLB 0:2 PD 1:0	DLB 79.5 PD 74	+	+	Sporadic DLB and PD	Ogaki et al., 2015
G4R (c.10G > A)	Heterozygous missense	MTS domain	Caucasian	0:1	69	+	+	DLB	Ogaki et al., 2015
S5R (c. 15C > G)	Heterozygous missense	MTS domain	Chinese	0:1	75	ND	ND	AD	Che et al., 2018
R8H (c. 23G > A)	Heterozygous missense	MTS domain	Japanese	1:0	38	+	+	Sporadic PD	Ikeda et al., 2017
P14S (c.40C > T)	Heterozygous missense	MTS domain	Caucasian	US 0:1	71	+	+	Sporadic PD	Ogaki et al., 2015
A16A (c.48C > T)	Heterozygous missense	MTS domain	Caucasian	US 1:0	60	+	+	Sporadic PD	Ogaki et al., 2015
R18Q (c.53G > A)	Heterozygous missense	MTS domain	Chinese	0:1	65	+	–	Late-onset sporadic PD	Yang et al., 2016
V31V (c.93C > T)	Heterozygous missense	MTS domain	Caucasian	0:1	65	+	–	Sporadic PD	Ogaki et al., 2015
A32T (c. 94G > A)	Heterozygous missense	MTS domain	Western European Ancestry	ND	US 45 FR 29.5	+	–	Early-onset PD	Jansen et al., 2015
			Chinese	0:1	70	–	–	AD	Che et al., 2018
P34L (c. 101C > T)	Heterozygous missense	MTS domain	Western European Ancestry	ND	US 45 FR 29.5	+	–	Early-onset PD	Jansen et al., 2015
			Caucasian	DLB 0:1 PD 1:1	DLB 90 PD 71	+	–	DLB and PD	Ogaki et al., 2015
A37V (c.110C > T)	Heterozygous missense	MTS domain	Caucasian	0:1	ND	–	+	DLB	Ogaki et al., 2015
A49V (c.146C > T)	Heterozygous missense	MTS domain	Caucasian	0:1	76	+	+	sporadic PD	Ogaki et al., 2015
T61I (c.182C > T)	Heterozygous missense	Conserved α-helix	Japanese	6:4	49.4	+	+	Late-onset autosomal dominant PD	Funayama et al., 2015
			Chinese	2:5	48.3	+	+	Early-onset autosomal dominant PD	Shi et al., 2016
V66M (c.196G > A)	Heterozygous missense	Conserved α-helix	Italian	1:0	60	+	+	MSA	Nicoletti et al., 2018
A71P (c.211G > C)	Homozygous missense	Conserved α-helix	Caucasian	1:0	26	+	+	Early-onset PD	Lee et al., 2018
A79S (c.235G > T)	Heterozygous missense	Conserved α-helix	Chinese	0:1	42	+	–	Sporadic PD	Yang et al., 2019
I80V	Heterozygous missense	Conserved α-helix	Western European Ancestry	ND	US 45 FR 29.5	+	–	Early-onset PD	Jansen et al., 2015
S85R (c. 255T > A)	Heterozygous missense	Disordered region	Chinese	1:0	65	+	+	Behavioral variant FTD	Che et al., 2018
A93V (c.278C > T)	Heterozygous missense	Disordered region	Caucasian	1:0	85	+	+	DLB	Ogaki et al., 2015
Q126X (c.376C > T)	Heterozygous nonsense	CHCH domain	German	0:1	>40	–	–	Early-onset PD	Koschmidder et al., 2016
R145Q (c. 434G > A)	Heterozygous missense	C-terminus	Japanese	1:0	67	+	–	Late-onset autosomal dominant PD	Funayama et al., 2015
			Chinese	1:0	41	+	+	Early-onset sporadic PD	Yang et al., 2016
c. *154A > G	SNV	3′UTR	Chinese	ND	ND	ND	ND	autosomal dominant PD	Wu et al., 2016
c. −9T > G	SNV	5′UTR	Japanese	ND	ND	ND	ND	Sporadic PD	Funayama et al., 2015
			Caucasian	ND	ND	ND	ND	DLB	Ogaki et al., 2015

*PD, Parkinson’s Disease; AD, Alzheimer’s Disease; MSA, Multiple System Atrophy; DLB, dementia with Lewy bodies; MTS, mitochondrial targeting sequence; UTR, untranslated region; FTD, frontotemporal dementia; SNV, single nucleotide variant; US, United States; FR, France; ND, not determined.*

Exome sequencing of another series of 1243 PD and 472 controls of western European ancestry identified A32T, P34L, and I80V rare variants of CHCHD2 in four early-onset PD patients and none in controls (Jansen et al., 2015). These mutations occur at highly conserved residues, two of which are in the MTS of CHCHD2 (Jansen et al., 2015). In addition, a nonsense mutation (Q126X) leading to truncation of the CHCHD2 protein was identified in an early-onset PD patient from Germany (Koschmidder et al., 2016). Intriguingly, a homozygous missense mutation in the highly conserved hydrophobic helix region of CHCHD2 (A71P) was found in a 26-year-old homozygous recessive early-onset PD patient (Lee et al., 2018). Indeed, the homozygous A71P mutation exhibited pathogenicity, as fibroblasts derived from this patient demonstrated fragmentation of the mitochondrial network, reduced oxidative phosphorylation activity in complexes I and IV, and increased ROS production (Lee et al., 2018). Finally, a screen of 27 patients with MSA in southern Italy identified the CHCHD2 V66M mutation in an MSA patient, a mutation that was absent in 500 healthy controls (Nicoletti et al., 2018).

Despite the presence of rare pathogenic CHCHD2 mutations in early-onset PD and other neurodegenerative diseases, other studies have failed to provide evidence for genetic association of CHCHD2 to PD. For example, only four non-coding variants of *CHCHD2* were found in a Spanish cohort of 536 PD and 518 unrelated controls, none of which were significantly associated with sporadic PD (Tejera-Parrado et al., 2017). A study from a Brazilian familial autosomal dominant PD cohort failed to find any pathogenic CHCHD2 mutations from 122 index cases (Voigt et al., 2019). Likewise, no pathogenic CHCHD2 mutations were found in southern Italian (Gagliardi et al., 2017) or Canadian (Zhang et al., 2016) cohorts of 165 and 155 autosomal dominant PD patients, respectively. While these negative results do not bear on the pathogenicity of known CHCHD2 mutations, they do collectively indicate that pathogenic CHCHD2 mutations are exceptionally rare and contribute to far less than 1% of the overall prevalence of PD and LBDs. The frequency and positions of CHCHD2 mutations appear to vary between different populations at the global scale. However, rare putative pathogenic CHCHD2 variants defined to select populations do exist, and these variants are greatly overrepresented in PD and LBD patients compared to controls in both Asian and Caucasian populations.

Studies of both Caucasian and Asian populations have reported the prevalence of motor and non-motor symptoms in their patients (Table 1). Motor symptoms include but are not limited to: resting tremor, bradykinesia, rigidity, gait disturbances, hyperreflexia, postural instability, and restless leg syndrome. Non-motor symptoms involve loss of smell or taste, hallucinations, dementia, depression, orthostatic hypotension, constipation, and urinary urgencies. Multiple patients where CHCHD2 mutations have been identified presented both motor and non-motor symptoms (P2L, G4R, R8H, P14S, A16A, A49V, T61I, and V66M) (Funayama et al., 2015; Ogaki et al., 2015; Shi et al., 2016; Ikeda et al., 2017; Nicoletti et al., 2018). Few patients presented motor symptoms only (R18Q, V31V, A32T, and P34L) and one patient showed non-motor symptoms only (A37A variant) (Jansen et al., 2015; Ogaki et al., 2015; Yang et al., 2016). Furthermore, these studies did not report sex differences in CHCHD2 mutations as a key factor.

## Physiological Functions of CHCHD2

### Bi-organellar Localization and Stress Response

**FIGURE 2:**
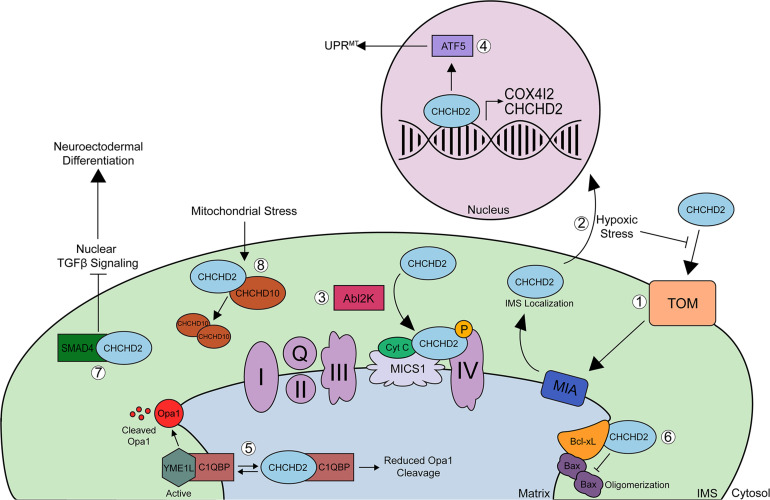
Schematic of CHCHD2 functions in mitochondria and nucleus. **(1)** Precursor, reduced CHCHD2 is imported to the IMS of mitochondria via the TOM channel in a reduced state. Once CHCHD2 is imported, CHCHD2 interacts with oxidoreductase, Mia40. Mia40 redox-coupled, thiol-disulfide exchange system, inserts disulfide bonds into CHCHD2, and CHCHD2 is localized IMS. **(2)** Under conditions of hypoxic or oxidative stress, mitochondrial import of CHCHD2 is suppressed, allowing CHCHD2 to accumulate in the nucleus, where it acts as a transcription factor for cytochrome c oxidase (COX) subunit 4 isoform 2 (COX412) and CHCHD2 itself. **(3)** In the mitochondrial IMS, CHCHD2 binds to cytochrome c, MICS1, and COX to regulate COX activity (complex IV). Phosphorylation of CHCHD2 by Abl2 kinase increases its affinity for COX, resulting in increased respiratory activity. **(4)** CHCHD2 promotes mitochondrial unfolded protein response (UPRmt) and mitochondrial biogenesis by activating ATF5. **(5)** CHCHD2 competes with YME1L for binding to C1qBP, thereby decreasing OPA1 degradation by YME1L and promoting normal mitochondrial morphology. The binding of CHCHD2 to C1qBP also suppresses the anti-cell migration activity of C1qBP, therby enhancing cell migration. **(6)** CHCHD2 enhances the ability of Bcl-xL to suppress pro-apoptotic Bax oligomerization, thereby inhibiting cytochrome c release. **(7)** The sequestration of SMAD4 by CHCHD2 to mitochondria suppresses TGFB signaling, which primes hiPSCs toward neuroectodermal differentiation. **(8)** Finally, mitochondrial stress and loss of mitochondrial membrane potential increases CHCHD2/CHCHD10 heterodimerization and CHCHD2 is required for the oligomerization of CHCHD10. The physiological role of homo-hetero oligomers of CHCHD2 and CHCHD10 remains to be elucidated.

Coiled-coil-helix-coiled-coil-helix domain containing 2 is an ∼18-kDa nuclear-encoded mitochondrial protein that has both mitochondrial and nuclear functions. CHCHD2 has been shown to be evenly expressed throughout various tissues in the body including the brain (cortex, midbrain, and striatum), spinal cord, spleen, and kidney and is highly expressed in tissues from the heart, liver, smooth muscle, lung, and pancreas (Burstein et al., 2018; Sato et al., 2021). While CHCHD2 shows strong expression in dopaminergic neurons of the substantia nigra pars compacta, midbrain, and pyramidal neurons in the hippocampus and cortex (Huang et al., 2018), it has not been explored whether CHCHD2 is expressed in glial cells. Under normal physiologic conditions, CHCHD2 is imported to the IMS of mitochondria via the Mia40 redox-coupled thiol-disulfide exchange system within the IMS (Figure 2; Aras et al., 2015). In the IMS, CHCHD2 plays a regulatory role in various cellular processes including oxidative stress response, cellular migration and differentiation, electron transport, mitochondrial morphology and cristae structure, and apoptosis. Cysteine mutations C134S or C144S within the CX_9_C domain impair the proper mitochondrial import of CHCHD2, and mutations in all 4 cysteines in CX_9_C completely block Mia40 binding and mitochondrial import of CHCHD2 (Aras et al., 2015). Under conditions of hypoxic or oxidative stress, however, mitochondrial import of CHCHD2 is inhibited probably by disrupting Mia40-CHCHD2 redox-coupling, allowing CHCHD2 to accumulate in the nucleus (Aras et al., 2013, 2015, 2020). In the nucleus, CHCHD2 acts as a transcription factor for cytochrome c oxidase (COX) subunit 4 isoform 2 (COX4I2) (Aras et al., 2013, 2015) and CHCHD2 itself (Figure 2; Aras et al., 2015). Increased CHCHD2 induces the mitochondrial unfolded protein response (UPR^*mt*^), autophagy, and mitochondrial biogenesis, thereby rescuing mitochondrial impairments in DW7 MELAS cells (Aras et al., 2015). CHCHD2 also promotes UPR^*mt*^ by positively regulating the ATF5 transcription factor (Figure 2; Aras et al., 2015). Under hypoxic conditions, COX4I2 and CHCHD2 levels are upregulated (Aras et al., 2013), which stabilizes oxidative phosphorylation (OXPHOS) and compensates for the lack of energy caused by ROS production (Liu and Zhang, 2015), thereby promoting mitochondrial respiration stabilization.

### Regulation of Cell Migration and Differentiation

Prior to the discovery of *CHCHD2* as a potential PD gene, an unbiased functional cDNA library screen identified CHCHD2 to contain cell migration-promoting activity through the activation of the Akt, RhoA/ROCK, and Jnk pathways in NIH3T3 cells (Seo et al.). Such action of CHCHD2 was shown to be achieved through its interaction with hyaluronic acid-binding protein 1 (HABP1) (Seo et al., 2010). However, whether this protein interaction occurs within mitochondria was not investigated in the study. The cell migration-promoting activity of CHCHD2 was also confirmed in human induced pluripotent stem cells (hiPSCs) (Zhu et al., 2016). In this study, the authors identified CHCHD2 as a key protein that primes hiPSCs for neuroectodermal differentiation by sequestering SMAD4 within mitochondria (Zhu et al., 2016). Mitochondrial sequestration of SMAD4 by CHCHD2 suppresses TGFβ nuclear signaling, thereby promoting neuroectodermal differentiation (Figure 2; Zhu et al., 2016). Interestingly, CHCHD2 is co-amplified with EGFR in non-small cell lung carcinoma (NSCLC), and knockdown of CHCHD2 in NSCLC not only reduces cell migration but also cell proliferation and mitochondrial respiration (Wei et al., 2015). Consistent with its mitochondrial and nuclear functions, the CHCHD2 hub interactome includes the mitochondrial protein C1qBP and the oncogenic transcription factor YBX1, as identified by unbiased affinity purification mass spectrometry (Wei et al., 2015). While other studies have independently validated the functional interaction of CHCHD2 with C1qBP (Seo et al., 2010; Liu W. et al., 2020), the functional consequences of CHCHD2-YBX1 interaction are unknown.

### Regulation of Mitochondrial OXPHOS

Within mitochondria, CHCHD2 regulates electron transport between the respiratory complexes (Palikaras et al., 2015). COX is involved in transferring electrons between complex III and complex IV, and CHCHD2 binds to COX, cytochrome c, and MICS1 to regulate COX activity and cytochrome c release (Figure 2; Oka et al., 2008; Meng et al., 2017). CHCHD2 undergoes phosphorylation at Y99 by the tyrosine kinase AbI2, thereby enhancing the interaction between COX and CHCHD2, resulting in increased respiratory activity (Figure 2; Aras et al., 2017). However, whether CHCHD10 undergoes phosphorylation at Y99 by AbI2 has not been studied (Aras et al., 2015). Interestingly, the decline of phosphorylation of CHCHD2 at Y99 was detected in CHCHD10 knockdown cells and the overexpression of Y99 phosphomimetic (Y99E) CHCHD2 can rescue the impaired mitochondrial oxygen consumption in CHCHD10 knockdown cells (Purandare et al., 2018). These data indicate that CHCHD2 and CHCHD10 closely cooperate to regulate mitochondrial respiratory complexes. Furthermore, it has been shown that loss of *CG5010*, the *Drosophila* ortholog of mammalian *CHCHD2* and *CHCHD10*, reduces ATP production, oxygen consumption rate (OCR), and spare respiratory capacity in *Drosophila* embryonic cells (Meng et al., 2017), suggesting that CG5010 maintains electron flow in the ETC from complexes I and II to complex IV in *Drosophila* (Meng et al., 2017). Interestingly, expression of wild type (WT) CHCHD2 in *CG5010* knockout cells suppresses cytochrome c release and therefore downstream caspase activation, while the PD-associated CHCHD2 T61I or R145Q mutants do not (Meng et al., 2017), indicating that T61I and R145Q are at least partial loss-of-function mutations in *Drosophila*. In PD patient fibroblasts harboring the homozygous CHCHD2 A71P mutation, CHCHD2 levels are significantly decreased along with subunits of complexes I, IV, and V, resulting in impaired respiratory rates of complex I and IV (Lee et al., 2018). These impairments are rescued by expression of exogenous WT CHCHD2 (Lee et al., 2018), suggesting that the homozygous A71P mutation is at least in part a loss-of-function mutation. PD patient fibroblasts also show significantly increased superoxide levels under normal glucose conditions, which is exacerbated when cultured with galactose (Lee et al., 2018). Such increased ROS are associated with reduced neuronal viability, particularly in DA neurons (Valencia and Moran, 2004; Hwang, 2013).

### Regulation of Mitochondrial Structure and Apoptosis

Coiled-coil-helix-coiled-coil-helix domain containing 2 is thought to be involved in the maintenance of mitochondrial cristae structure via its interaction with OPA1, a protein that mediates mitochondrial inner membrane fusion in response to mitochondrial stress (Liu W. et al., 2020; Liu Y.T. et al., 2020), and its interaction with MICS1, an inner membrane protein that regulates mitochondrial morphology and cytochrome c release (Meng et al., 2017; Lee et al., 2018). The interaction between C1qBP/p32 and YME1L, a protease that degrades OPA1, strengthens the ability of YME1L to degrade OPA1 (Liu W. et al., 2020; Liu Y.T. et al., 2020). CHCHD2 competes with YME1L for binding to C1qBP/p32, thereby decreasing OPA1 degradation and promoting normal mitochondrial morphology (Figure 2; Liu W. et al., 2020). Reduced CHCHD2 leads to decreased OPA1 levels, as OPA1 becomes degraded by YME1L, leading to fragmented mitochondria (Liu W. et al., 2020). Furthermore, under stress conditions, the OMA1 peptidase cleaves OPA1, resulting in fragmented mitochondria and abnormal cristae structure (Liu Y.T. et al., 2020). In *Drosophila CG5010* knock out animals, expression of WT CHCHD2 rescues mitochondrial cristae abnormalities, whereas expression of CHCHD2 T61I or R145Q mutations failed to rescue such cristae defects (Meng et al., 2017). Likewise, expression of WT CHCHD2 in *Drosophila CG5010* knockout cells suppresses cytochrome c release and therefore downstream caspase activation, while the PD-linked CHCHD2 T61I or R145Q mutants do not (Meng et al., 2017), collectively indicating that T61I and R145Q are at least partial loss-of-function mutations in *Drosophila*. Such CHCHD2-mediated phenotypes are not only associated with the ability of CHCHD2 to bind to cytochrome c and MICS1 (Meng et al., 2017; Lee et al., 2018), but also its interaction with Bcl-xL (Liu et al., 2015). Under physiological conditions, CHCHD2 binds to the anti-apoptotic protein Bcl-xL and enhances the ability of Bcl-xL to suppress pro-apoptotic Bax oligomerization and its mitochondrial localization (Figure 2; Liu and Zhang, 2015; Liu et al., 2015). In the presence of apoptotic stimuli, loss of CHCHD2 in mitochondria attenuates the ability of Bcl-xL to suppress Bax, thereby enhancing cytochrome c release and apoptosis (Liu and Zhang, 2015; Liu et al., 2015). These data collectively indicate that CHCHD2 plays a role as an anti-apoptotic protein within mitochondria (Liu et al., 2015).

## PD, CHCHD2, and α-Synuclein Pathology

Mitochondrial dysfunction is a major hallmark of neurodegenerative disease, including synucleinopathies, such as MSA, PD, PDD, and DLB. Over the past 47 years, human and animal studies have shown that the mitochondrial toxin 1-methyl-4-phenyl-1,2,3,6-tetrahydropyridine (MPTP), a mitochondrial complex I inhibitor, induces parkinsonian-like symptoms associated with the loss of DA neurons (Langston et al., 1983; Kopin and Markey, 1988; Kopin, 1992; Langston, 2017; Mat Taib and Mustapha, 2020). Such studies have cemented the link between mitochondrial dysfunction and PD. In 1989, complex I activity in the mitochondrial ETC was found to be significantly reduced in PD patients (Parker et al., 1989), with complexes II and III at slightly lower activity and complex IV not being affected (Parker et al., 1989; Chagnon et al., 1995). Further, complex I activity is decreased in PDD patients, indicating that mitochondrial dysfunction may also contribute to non-motor PD symptoms (Gatt et al., 2016). These findings, together with the discovery of α-synuclein as the major component of Lewy bodies (Polymeropoulos et al., 1997; Braak et al., 2004), jumpstarted the search for connections between α-synuclein and mitochondria.

Later studies have indeed demonstrated pathologically relevant connections between mitochondrial dysfunction and α-synuclein. Specifically, genetic loss of α-synuclein significantly attenuates the loss of tyrosine hydroxylase-positive DA neurons and PD-like symptoms upon MPTP exposure (Dauer et al., 2002; Schluter et al., 2003), and MPTP treatment reciprocally exacerbates Lewy body pathology in mutant α-synuclein transgenic models (Nieto et al., 2006; Yu et al., 2008). Di Maio et al. (2016) discovered that α-synuclein binds to Tom20, a mitochondrial outer membrane protein that plays a key role in protein import. Interestingly, only oligomeric, dopamine-modified, and S129E phospho-mimetic forms of α-synuclein inhibit mitochondrial import by disrupting Tom20 (Di Maio et al., 2016), and monomeric and nitrated forms of α-synuclein are unable to inhibit import. These data suggest that only specific pathogenic forms of the protein are capable of blocking import through Tom20 (Di Maio et al., 2016). Another study also showed that S129 phospho-α-synuclein preferentially binds to mitochondria in both primary neurons and post-mortem brain tissue (Wang et al., 2019), again highlighting the role of pathologically relevant forms of α-synuclein accumulating in mitochondria.

Despite multiple connections between α-synuclein and mitochondrial pathogenesis, the mechanistic basis for the role of CHCHD2 and α-synucleinopathy still remains unclear, albeit with a few tantalizing observations. Specifically, α-synuclein interacts with cytochrome c, a mitochondrial protein located in the IMS (Hashimoto et al., 1999; Kumar et al., 2016; Meng et al., 2017) that transfers electrons between complexes III and IV, which upon release into the cytosol can trigger apoptosis (Liu et al., 1996; Perez-Mejias et al., 2019). Cytochrome c release induces the oligomerization of α-synuclein and promotes radical formation, thereby contributing to mitochondrial dysfunction and disease progression (Kumar et al., 2016). Furthermore, a recent study has shown that while oxidized cytochrome c interacts with α-synuclein to inhibit its aggregation by favoring a hetero-dityrosine bond formation, reduced cytochrome c interacts with α-synuclein to facilitate its aggregation (Ghosh et al., 2019).

As CHCHD2 also interacts with cytochrome c and CHCHD2 dysfunction (loss of CHCHD2 or PD-linked mutations) increases cytochrome c release (Meng et al., 2017), such CHCHD2-mediated cytochrome c release may also drive increased α-synuclein oligomerization and aggregation. Furthermore, it has been shown that loss of CHCHD2 decreases ATP production, increases mitochondrial peroxide production, and lowers Ca^2+^-buffering activity in DA terminals in *Drosophila* (Imai et al., 2019a). Interestingly, these pathogenic signatures caused by CHCHD2 deficiency were rescued by the light-dependent activation of mitochondrion targeted dR (mito-dR) (Imai et al., 2019a).

While such a proposed mechanism is generic and simplistic, post-mortem assessment of brain tissues from a PD patient with the heterozygous CHCHD2 T61I mutation demonstrate the accumulation of soluble and insoluble S129 phospho-α-synuclein, as well as increased insoluble CHCHD2 and CHCHD10 (Ikeda et al., 2019). By utilizing patient-derived iPSCs and *Drosophila* models, the same study found that sarkosyl-insoluble α-synuclein is increased in DA neurons derived from iPSCs expressing the CHCHD2 T61I mutation (Ikeda et al., 2019). Higher levels of LC3B-II and cleaved caspase 3 are also observed (Ikeda et al., 2019), indicating changes in autophagy and cell death vulnerability, respectively. Further, characterization of α-synuclein fibrils from this patient also shows similarities in α-synuclein species from patients with *SNCA* duplication, DLB, and CHCHD2 T61I mutation (Ikeda et al., 2019).

## Animal Models of CHCHD2

### *C. elegans* Models

*Caenorhabditis elegans* is a nematode worm known for its simplistic nervous system and ease of detecting movement changes. *C. elegans* contains ∼1,000 somatic cells with defined tissues such as muscle, intestine, and skin (Edgar and Wood, 1977). *C. elegans* is a powerful genetic model for understanding the molecular mechanisms of neurodegenerative diseases and understanding how molecular changes can lead to motor phenotypes. Furthermore, *C. elegans* models offer advantages to study mitochondrial function because mitochondrial proteins between *C. elegans* and mammals are highly conserved, and the function and structure of mitochondrial respiratory chains are also highly similar (Murfitt et al., 1976). *Har-1* is the ortholog of both *CHCHD2* and *CHCHD10* in *C. elegans*, with nearly equal amino acid similarity to both. *Har-1^–/–^* nematodes exhibit slower rates of movement, a curling phenotype, and shortened lifespan, associated with mitochondrial superoxide overproduction compared to wild type N2 *C. elegans* (Woo et al., 2017). Expression of CHCHD10 but not FTD/ALS-linked CHCHD10 mutants (S59L and R15L) in *har-1^–/–^ C. elegans* rescues all pathogenic phenotypes (Woo et al., 2017). While it is likely that CHCHD2 expression in *har-1^–/–^ C. elegans* will also rescue such phenotypes, this experiment has yet to be reported. *Har-1* was originally found in a drug resistance screen to an antimitotic drug, hemiasterlin, from which the *har-1* G73E mutant was identified (Zubovych et al., 2010). However, unlike *har-1^–/–^* animals (Woo et al., 2017), *har-1* G73E mutants exhibit normal movement and lifespans but reduced sensitivity to hemiasterlin (Zubovych et al., 2010).

### *Drosophila* Models

In *Drosophila, CG5010* is the ortholog of both *CHCHD2* and *CHCHD10* in mammals. Like in *C. elegans* (Woo et al., 2017), knocking out *CG5010* in *Drosophila* shortens lifespan, reduces ATP production, and disrupts mitochondrial cristae structure, suggesting that CG5010 plays a role in maintaining cristae organization and function (Meng et al., 2017; Imai et al., 2019a; Liu W. et al., 2020). Furthermore, loss of *CG5010* in *Drosophila* embryonic cells reduces ATP production, OCR, and spare respiratory capacity (Meng et al., 2017). These data indicate that CG5010 maintains electron flow in the ETC from complexes I and II to complex IV in *Drosophila* (Meng et al., 2017). Interestingly, expression of exogenous human or *Drosophila* CHCHD2 rescued cristae abnormalities and respiration defects in *CHCHD2*^–/–^ mouse embryonic fibroblasts and hypomorphic dCHCHD2 H43 in *Drosophila* (Meng et al., 2017). However, the expression of PD-associated mutations T61I or R145Q failed to suppress the pathogenic phenotypes (Meng et al., 2017). Furthermore, *CG5010*^–/–^ flies exhibit reduced climbing abilities and tyrosine hydroxylase (TH) signals, indicating a decline in DA neuron activity or survival (Meng et al., 2017; Liu W. et al., 2020). Additionally, loss of *CG5010* increases susceptibility to oxidative stress from peroxide and paraquat exposure by overwhelming molecular ROS scavengers (Meng et al., 2017).

Overexpression of PD-linked CHCHD2 T61I, R145Q, or P2L mutations lead to locomotor impairments in 30 to 40-day-old flies (Tio et al., 2017). Interestingly, not only PD-linked CHCHD2 mutations but also human WT CHCHD2-expressing *Drosophila* showed climbing impairments after aging, suggesting that excessive WT CHCHD2 may also be detrimental in flies (Tio et al., 2017). In addition, the severity of the climbing phenotype is exacerbated by the complex I inhibitor rotenone in CHCHD2 T61I and R145Q-expressing flies, but not in WT or P2L-expressing flies (Tio et al., 2017). Furthermore, while CHCHD2 WT, T61I, R145Q, and P2L variants significantly induce DA neuron degeneration and shortened life span, T61I and R145Q variants show the highest mortality rates among CHCHD2 variants, indicating that T61I and R145Q mutants exhibit more severe phenotypes than either WT or P2L in *Drosophila* (Tio et al., 2017).

Overexpression of α-synuclein together with the loss of *CG5010* synergistically worsens climbing defects, shortens lifespan, and promotes the loss of DA neurons (Ikeda et al., 2019). Expression of the PD-linked mutant CHCHD2 T61I with α-synuclein also synergistically exacerbates survival and motor deficits (Ikeda et al., 2019), demonstrating pathological synergy between α-synuclein and the CHCHD2 T61I mutation. Polyubiquitin signals accumulate in *CG5010*^–/–^ flies with insoluble α-synuclein, which correlates with increased p62 levels (Ikeda et al., 2019). Similar results are seen with overexpression of CHCHD2 T61I, suggesting that CHCHD2 dysfunction (loss of CHCHD2 or CHCHD2 mutations) and α-synuclein may synergistically deregulate the ubiquitin-proteasome system and autophagy (Ikeda et al., 2019).

### Mouse Models

As mammals express both *CHCHD2* and *CHCHD10*, genetic loss of either gene produces subtle to no phenotype (Burstein et al., 2018; Liu Y.T. et al., 2020; Ryan et al., 2021; Sato et al., 2021), likely due to redundancy and compensation. However, it was recently reported that *CHCHD2*^–/–^ mice show p62 inclusion formation and dopaminergic neuronal loss in an age-dependent manner (Sato et al., 2021). In this study, the authors have shown that *CHCHD2*^–/–^ mice are viable and indistinguishable in appearance at birth compared to control. However, 2-year-old *CHCHD2*^–/–^ mice exhibit fragmented mitochondria in DA neurons, abnormal motor phenotypes, loss of DA neurons in the substantia nigra, and the formation of p62 inclusions (Sato et al., 2021), suggesting a lack of compensation with age. However, double knockouts of both *CHCHD2* and *CHCHD10* (DKO) display disrupted mitochondrial cristae structure, reduced mitochondrial respiration, and increased OPA1 cleavage by OMA1 (Liu Y.T. et al., 2020). The DKO animals appear to exhibit mitochondrial phenotypes, including OPA1 cleavage reminiscent of the FTD/ALS-linked CHCHD10 S59L mutation (Liu T. et al., 2020; Liu Y.T. et al., 2020). In addition, DKO mice exhibit cardiomyopathy associated with increased methylenetetrahydrofolate dehydrogenases (MTHDFDs) and activation of the integrated mitochondrial stress response (Liu Y.T. et al., 2020), similar to that observed in CHCHD10 S59L knock-in mice (Anderson et al., 2019; Genin et al., 2019).

Despite the characterization of *C. elegans* and *Drosophila* models of CHCHD2, no transgenic or knock-in models of CHCHD2 mutations have been reported in mice. However, such models are reportedly being developed and characterized. Coupled with other mammalian models of PD (α-synuclein, PINK1, Parkin, MPTP, etc.), these new mouse models of CHCHD2 could provide relevant insight into Lewy body pathology and other disease-associated motor and behavioral phenotypes in mammalian systems. CHCHD2 mouse models that can accurately capture the pathology seen in patients would greatly assist in determining the molecular consequences of CHCHD2 dysfunction and how it relates to LBDs.

## Toward Understanding the Divergent Functions of CHCHD2 and CHCHD10

*CHCHD2* and *CHCHD10* diverged from a common ancestral gene, as evidenced by a single orthologous gene in *Drosophila*, *C. elegans*, and *Saccharomyces cerevisiae*. Major functions of CHCHD10, CHCHD2, *C. elegans* har-1, *Drosophila* CG5010, and *S. cerevisiae* Mix17/Mic17, proteins are also conserved, as human CHCHD10 completely rescues the phenotypes associated with *har-1^–/–^* (Woo et al., 2017), and human CHCHD2 rescues phenotypes associated with *CG5010*^–/–^ (Cavallaro, 2010; Meng et al., 2017). In mammals, both CHCHD2 and CHCHD10 are small (15–18 kDa) proteins located in the mitochondrial IMS (Bannwarth et al., 2014; Liu et al., 2015), which share 54% amino acid sequence identity (Imai et al., 2019b). Both CHCHD2 and CHCHD10 share similar expression patterns in tissues throughout the body (Burstein et al., 2018). Neither single knockout of CHCHD10 nor CHCHD2 exhibits a salient phenotype (Burstein et al., 2018; Liu Y.T. et al., 2020), whereas double knockouts exhibit clear phenotypes (Liu Y.T. et al., 2020), indicating redundancy and compensation for the loss of each other. However, this compensation may be less effective with age (Sato et al., 2021). The relationship between CHCHD2 and CHCHD10 is further complicated by the ability of CHCHD2 and CHCHD10 to form homodimers and heterodimers with each other (Figure 2; Meng et al., 2017; Burstein et al., 2018; Huang et al., 2018; Straub et al., 2018). Both CHCHD2 and CHCHD10 prevent the production of ROS, maintain cristae structure, and can translocate to the nucleus in response to stress (Burstein et al., 2018; Purandare et al., 2018; Straub et al., 2018). While CHCHD2 and CHCHD10 share significant similarities in sequence, secondary structure, expression pattern, and function, mutations in CHCHD2 and CHCHD10 are associated with distinctly different neurodegenerative diseases. CHCHD2 mutations are associated with the spectrum of LBDs (Funayama et al., 2015), while CHCHD10 mutations are associated with the spectrum of FTD-ALS and mitochondrial myopathies (Bannwarth et al., 2014; Johnson et al., 2014; Müller et al., 2014; Ajroud-Driss et al., 2015; Zhang et al., 2015; Genin et al., 2016; Rubino et al., 2018). Previous studies have evaluated the prevalence of *CHCHD2* and *CHCHD10* mutations in Italian mitochondrial myopathy patients without mitochondrial DNA mutations (Rubino et al., 2018). In this study, the authors did not find novel pathogenic *CHCHD2* mutations in mitochondrial myopathy patients using morphological, biochemical, and molecular biopsies. However, the CHCHD10 homozygous P96T substitution was found in 1 sporadic mitochondrial myopathy patient (Rubino et al., 2018). A new study by Ryan et al. has shown that ALS-linked CHCHD10 R15L transgenic mice exhibit age-correlated pathologies in skeletal muscle, the CNS, and the heart. Interestingly, CHCHD10 R15L transgenic mice die from cardiac failure because of the degeneration of cardiomyocytes. Despite these pathologies, CHCHD10 R15L transgenic mice do not exhibit paralysis or abnormal motor phenotypes (Ryan et al., 2021). Perhaps the divergent disease phenotypes between CHCHD2 mutations and CHCHD10 mutations may in part be explained by their ability to associate with different pathological proteins such as α-synuclein (Ikeda et al., 2019; Imai et al., 2019a) and TDP-43 (Woo et al., 2017; Genin et al., 2019; Liu T. et al., 2020), respectively, the latter of which is a pathological hallmark of FTD-ALS (Neumann et al., 2006). However, few studies have directly compared CHCHD2 and CHCHD10 mutations on various pathological or mitochondrial phenotypes in the same study, making conclusive comparisons of biological or pathological activity difficult. The comparisons between CHCHD10 and CHCHD2 in terms of differential expression in specific types of cells, function, and binding to cytochrome c have not been systematically investigated. Hence, the underlying cause for such pathological divergence is still largely a mystery. Future studies directly comparing the mechanistic basis of CHCHD2 and CHCHD10 mutations driving distinct pathological processes, particularly in animal models, will be key to understanding the nature of these disease-linked mutations.

## Concluding Remarks

Recent studies have linked mutations in CHCHD2 with the spectrum of LBDs. Overall, the frequency of CHCHD2 mutations in LBDs is extremely rare and vary between demographic groups in Asian and Caucasian populations (Foo et al., 2015; Funayama et al., 2015; Ogaki et al., 2015; Puschmann et al., 2015; Koschmidder et al., 2016; Li et al., 2016; Shi et al., 2016; Wu et al., 2016; Gagliardi et al., 2017; Tejera-Parrado et al., 2017; Nicoletti et al., 2018). The most convincing evidence for a genetic link between CHCHD2 and PD comes from the original study of a Japanese pedigree of PD carrying the heterozygous CHCHD2 T61I mutation (Funayama et al., 2015), from which 1 patient has come to autopsy. This patient indeed exhibits substantia nigra degeneration and Lewy body pathology (Ikeda et al., 2019). Interestingly, substantial amyloid plaque and neurofibrillary tangle pathology are also observed in this patient (Ikeda et al., 2019), suggesting that the CHCHD2 T61I mutation may drive pathologies beyond Lewy bodies. Despite these confirmatory observations in human, one patient is clearly not sufficient to conclude a causal pathological link between CHCHD2 and LBDs.

Both *Drosophila* and mammalian cell culture model systems, including patient-derived fibroblasts (Lee et al., 2018; Mao et al., 2019) and iPSCs (Zhu et al., 2016; Wang et al., 2018; Ikeda et al., 2019; Harjuhaahto et al., 2020), have demonstrated a clear role of CHCHD2 in mitochondrial homeostasis, including ETC regulation (Aras et al., 2013, 2017; Liu and Zhang, 2015; Palikaras et al., 2015; Meng et al., 2017), cytochrome c release (Liu et al., 2015; Meng et al., 2017), cristae structure maintenance (Meng et al., 2017; Lee et al., 2018; Liu W. et al., 2020; Liu Y.T. et al., 2020), and anti-apoptotic activity (Liu and Zhang, 2015; Liu et al., 2015), whereas several PD-linked mutations (T61I, R145Q, and A71P) generally lack or oppose the normal functions of CHCHD2 (Meng et al., 2017; Tio et al., 2017; Ikeda et al., 2019). While the role of CHCHD2 in mitophagy still remains to be investigated, it has been shown that the expression of CHCHD2 was increased in PINK1-and Parkin-deficient *Drosophila* (Meng et al., 2017). Interestingly, the loss of *CG5010* does not exacerbate the mitochondrial phenotype in flies with Parkin or PINK1 deficiency (Meng et al., 2017). Nonetheless, overexpression of PINK1 or Parkin in flies deficient in *CG5010* does lead to more robust changes in mitochondrial morphology, suggesting crosstalk between CHCHD2 and the PINK1-Parkin mitophagy pathway (Meng et al., 2017).

Experimental progress linking CHCHD2 mutations with α-synuclein pathology in *Drosophila* models has been encouraging (Ikeda et al., 2019). However, it is notable that heterologous expression of wild type human CHCHD2 also induces a degenerative phenotype in *Drosophila* (Tio et al., 2017), suggesting that *Drosophila* may not be an ideal organism to model human CHCHD2 mutations. Hence, there is a growing need for *in vivo* mammalian models of CHCHD2 mutations that faithfully recapitulate disease seen in human patients. While the constitutive *CHCHD2/CHCHD10* double knockout mouse model is highly informative (Liu Y.T. et al., 2020), it is imperative to create and characterize disease-linked CHCHD2 mutations in mammalian models. Hence, mouse models of human WT and mutant CHCHD2, whether by knock-in or targeted expression in specific neuronal populations, are paramount. These mouse models will be critical for validating pathological phenotypes observed in *Drosophila* and cellular models as well as human patients carrying CHCHD2 mutations. Understanding the pathophysiological implications of CHCHD2 mutations *in vivo* will not only shed light on the mechanisms underlying PD and other synucleinopathies, but also provide therapeutic interventions for PD and other Lewy body disorders.

## Author Contributions

All authors listed have made a substantial, direct and intellectual contribution to the work, and approved it for publication.

## Conflict of Interest

The authors declare that the research was conducted in the absence of any commercial or financial relationships that could be construed as a potential conflict of interest.
